# Development and validation of in-situ and laboratory X-ray fluorescence (XRF) spectroscopy methods for moss biomonitoring of metal pollution

**DOI:** 10.1016/j.mex.2021.101319

**Published:** 2021-03-27

**Authors:** Mathis L. Messager, Ian P. Davies, Phillip S. Levin

**Affiliations:** a School of Environmental and Forest Sciences, University of Washington, 3714 Garfield Place NE, 98195 Seattle, Washington, United States; b Department of Geography, McGill University, Burnside Hall Building, 805 Sherbrooke Street West, Montreal, QC H3A 0B9, Canada; c National Institute for Agricultural and Environmental Research (INRAE), 5 rue de la Doua, CS 20244, Villeurbanne Cedex 69625, France; d The Nature Conservancy, 74 Wall Street, 98121 Seattle, Washington, United States

**Keywords:** ICP-OES, pXRFXRF, Bryophyte, Pellet, Cu, Pb, Zn, Urban pollution, Air pollution, Heavy metals

## Abstract

Metals are among the pollutants of highest concern in urban areas due to their persistence, bioavailability and toxicity. High concentrations of metals threaten aquatic ecosystem functioning and biodiversity, as well as human health. High-resolution estimates of pollutant sources are required to mitigate exposure to toxic compounds by identifying the specific locations and associated site characteristics where the deposition of metals is greatest. Mosses have been widely used as low-cost biological monitors of metal pollution for decades, because they readily accumulate pollutants over time, reflecting long term pollution levels. However, spectroscopic techniques to determine the concentration of metal pollutants in moss samples still require expensive instrumentation and involve time consuming sample preparation protocols with heavy use of reagents. Here we present protocols to perform *in-situ* and laboratory X-ray fluorescence (XRF) spectroscopy of epiphytic moss as rapid, low-cost, and accurate alternatives to conventional metal pollution biomonitoring. We also report on a preliminary validation of the measurements using mass fractions determined by inductively coupled plasma atomic emission spectroscopy (ICP-OES) as reference.

• XRF measurements are taken from moss directly on tree trunks in less than five minutes.
• Grinding and pelletizing of moss enables definitive quantitation (R^2^>0.90) of metals through portable XRF.

## Graphical abstract

**Figure:**
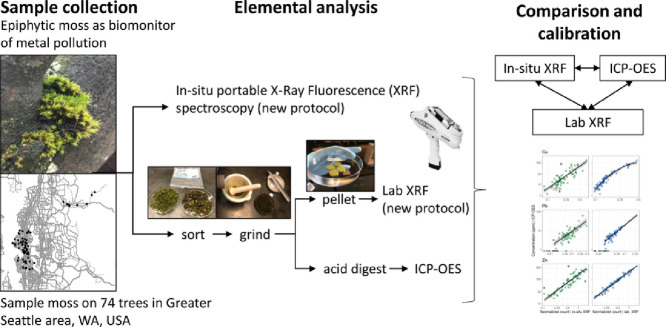

**Table:** Specifications Table

**Subject Area**	Environmental Science
**More specific subject area**	Biomonitoring, X-Ray Fluorescence Spectroscopy, Urban pollution
**Method name**	X-Ray Fluorescence Spectroscopy
**Name and reference of original method**	**Portable X-Ray Fluorescence Spectroscopy** • Bueno Guerra, M. B. et al. Comparison of analytical performance of benchtop and handheld energy dispersive X-ray fluorescence systems for the direct analysis of plant materials. *J. Anal. At. Spectrom.* **29**, 1667–1674 (2014). • Towett, E. K., Shepherd, K. D. & Lee Drake, B. Plant elemental composition and portable X-ray fluorescence (pXRF) spectroscopy: Quantification under different analytical parameters. *X-Ray Spectrom.* **45**, 117–124 (2016). • Queralt, I., Ovejero, M., Carvalho, M. L., Marques, A. F. & Llabrés, J. M. Quantitative determination of essential and trace element content of medicinal plants and their infusions by XRF and ICF techniques. *X-Ray Spectrom.* **34**, 213–217 (2005). • United States Environmental Protection Agency. *Field portable X-ray fluorescence spectrometry for the determination of elemental concentrations in soil and sediment. SW-846 Test Method 6200*. (2007). **Moss biomonitoring of metal pollution** • Fernández, J. A., Boquete, M. T., Carballeira, A. & Aboal, J. R. A critical review of protocols for moss biomonitoring of atmospheric deposition: Sampling and sample preparation. *Science of the Total Environment* **517**, 132–150 (2015).
**Resource availability**	

## Methods details

### Background

Biological monitoring provides a cost-effective alternative to automated stations commonly used for monitoring air quality when long-term, rather than daily, air pollution measurements are of interest [10,23]. Mosses in particular have been shown to be effective biological monitors of metal air pollution because they readily accumulate pollutants over time, reflecting long term pollution levels [8,16]. Such a low-cost alternative enables fine scale spatial assessments of metal pollution levels needed to calibrate high-resolution predictive models (e.g. companion paper [18]) and to support environmental justice studies [7].

Moss biomonitoring studies to date have relied on a variety of spectroscopic techniques to determine the concentration of metal pollutants in moss samples, including Inductively coupled plasma mass spectrometry (ICP-MS), optical emission spectrometry (ICP-OES), cold vapor atomic fluorescence or absorption spectrophotometry (CVAFS or CVAAS), electrothermal atomic absorption spectrometry (FAAS), and instrumental neutron activation analysis (INAA), among others [1,^11^,15]. These methods are expensive both in terms of fixed (i.e. machinery) and marginal (i.e. reagents) cost, often involving time consuming sample preparation protocols with heavy use of acids for digestion [9]. To the authors’ knowledge, no study has made use of energy-dispersive X-Ray Fluorescence (XRF) spectrometry in moss biomonitoring so far. Yet owing to new developments in XRF technology over the past decade, this method has been increasingly utilized to determine metal concentrations in leaves and other vascular plant material [17,20].

XRF relies on the emission of photons from an X-ray tube using Rh, Ag, Mo, Cr, or W as an anode [20]. The emitted photons interact with (excite) the atoms within the moss sample, which in turn emit photons (fluoresce) at energy levels that are specific to each element. Photons from the sample atoms are then received, counted, and converted by the instrument into a spectrum showing the number of counts per unit of time at a given energy level with identifiable peaks whose size reflects the relative quantity of each element in the sample [21].

XRF analysis techniques enable portable, rapid and low-cost analysis of multiple metallic elements at once, and imply simpler sample preparation with little to no reagent usage [17], potentially leading to an even greater reduction in the cost of moss biomonitoring compared to standard air quality station networks. XRF instruments are available in both benchtop and portable formats (pXRF), the latter allowing *in-situ* non-destructive analysis [5,20]. Here we present protocols to perform *in-situ* and laboratory (pellet-based) analysis of metal concentrations in moss with a portable XRF instrument. We also report on a preliminary validation of the measurements with ICP-OES analysis. See Messager et al. [18] for additional details about the corresponding study context and design.

### Study context

**Fig. 1:**
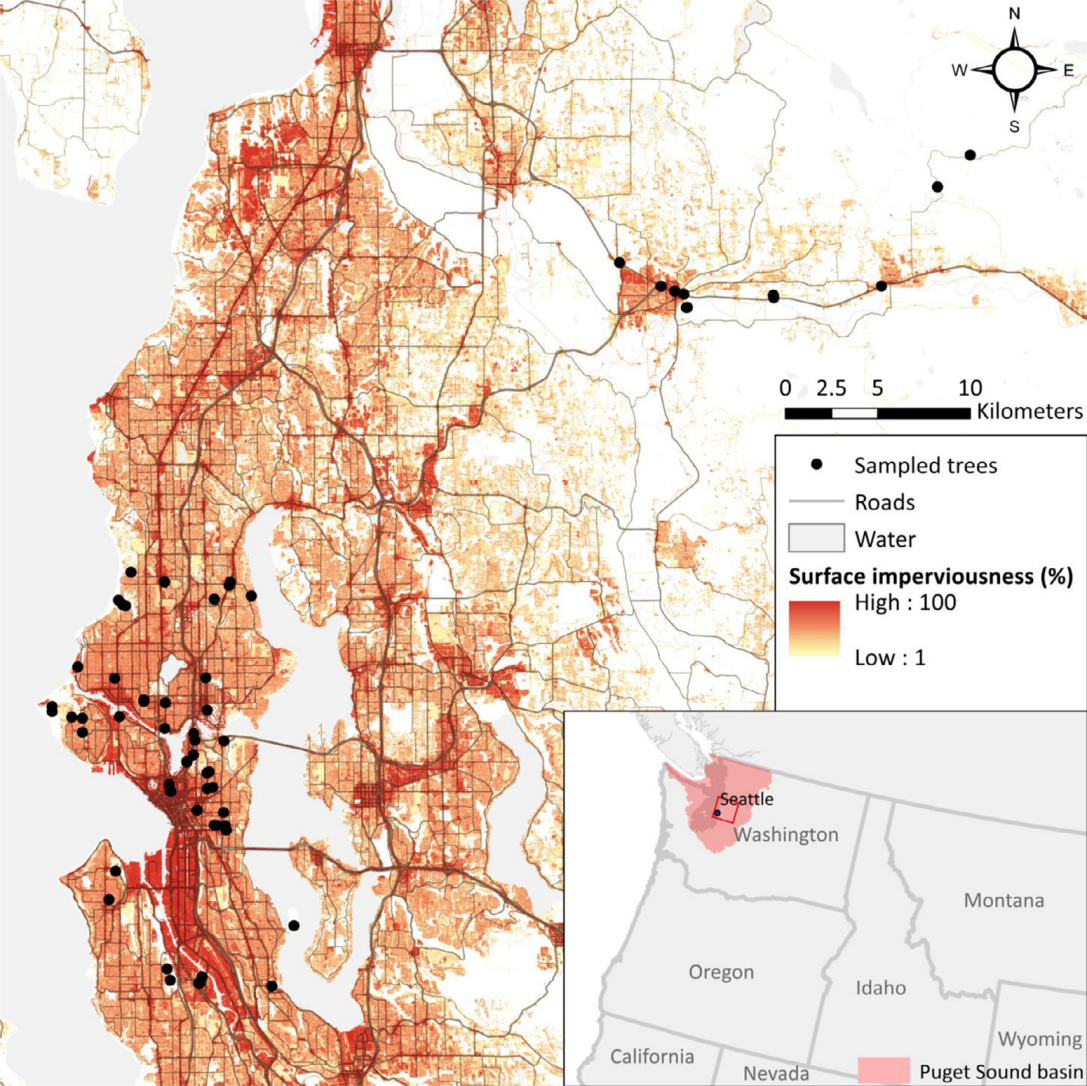
Spatial distribution of sampled trees used in methods validation (black points, *n* = 74), roads (gray lines, excluding local roads) and surface imperviousness (National Land Cover Dataset 2016) across the Greater Seattle area (Washington State, USA), the most densely urbanized region of the Puget Sound watershed (pink polygon in inset map).

Epiphytic moss (*Orthotrichum lyelli* Hook. & Taylor) samples were collected from 74 trees across 56 sites (mostly *Acer macrophyllum*) spread across a gradient of exposure to metal pollution through the Greater Seattle area, WA, USA (Fig. 1). *Orthotrichum lyelli* is a common bryophyte that grows on hardwood trees across the west coast of North America, Europe and North Africa [22] and was previously used in a biomonitoring study of urban air pollution in Portland, OR, USA [10]. Measurements were taken on moss from two closely adjacent trees (within 5–25 m of each other) at approximately one third (19 out of 56) of the sites, and from one tree in the rest of the sites. Through these near-duplicate samples, we aimed to capture small-scale variations in measured concentrations, and hence the reliability of these methods to assess site-scale pollution levels. Near-duplicate sampling was conducted at no more than one third of the sites to balance the number of sites where two trees were sampled with the total number of sampled sites. Sampling took place in July-August 2018.

### XRF instrumentation

All XRF assays (whether *in-situ* or in laboratory) were obtained using a portable XRF analyzer, the Bruker Tracer™ III-SD (T3S2606, Bruker Elemental, Kennewick, WA, USA; 4 W Rhodium anode and Silicon Drift Detector with 2048 channels) outfitted with a 4-µm thick protective Ultralene™ gridded window (P/N 485315–400, Bruker Kennewick, WA, USA) and a yellow filter (0.001″ Ti, 0.012″ Al). Differences in results between *in-situ* and laboratory measurements thus stem from manipulation and homogenization of the samples prior to assaying, rather than variations in precision/accuracy between benchtop and portable XRF instruments (contrary to e.g. [5]). The use of a yellow filter (and sufficient voltage) is the standard approach to measure the composition of modern alloys as it efficiently measures K-shell x-ray emission lines from Ti to Ag and L-shell lines from W to Bi. However, there is little sensitivity to elements below Ca with these settings. Although the primary goal of this study was to measure the relative concentration of Cu, Pb, and Zn, here we report method validation results for all elements detected by either XRF or ICP-OES analysis. The spot size of analysis was approximately 10 mm in diameter.

### Field measurements (*in-situ* XRF)

XRF measurements were taken from three different moss mats on each tree, all located at least one meter off the ground to avoid road spray and pet-related contaminants. For each tree, we assayed moss mats oriented in various directions (e.g. northward, southward from trunk) to capture microscale variations in contamination due to the spatial configuration of nearby pollutant sources (e.g. road) relative to the tree and dominant wind patterns. Measurements were only taken from mats large enough to fully cover the instrument's measurement window. No measurements were taken from dried out moss (brown-black rather than green in color).

After inspecting each moss mat to ensure that no branch or protruding bark could damage the instrument and verifying that the protective Ultralene gridded window on the instrument's measurement window was intact, the nozzle of the instrument was placed directly against the moss mat until the mat was well compacted against the measurement window. Spectra were acquired for 60 s per assay using a voltage of 40 keV, an anode current of 25 µA and no vacuum. All measurements were taken with powder-free nitrile gloves and the instrument nozzle was wiped with disposable non-abrasive lint-free paper tissues before every measurement.

### Field sampling collection (laboratory XRF)

Moss samples were collected from the same trees after taking *in-situ* measurements. Wearing powder-free nitrile gloves, a total of approximately 30 g (dry weight) of moss was collected from 3 to 10 mats (depending on the extent of moss coverage on the tree), including the same mats as those used for *in-situ* measurements (yet avoiding the patch within the mats which were compacted by the instrument).

Moss material was immediately placed in 18 oz (~500 mL) sterile polyethylene sampling bags with flat-wire closures, sealed, and stored by the end of the day at 4°C until analysis. Note that the precise amount of material required to subsequently make pellets or conduct ICP-OES analysis decreases with the relative amount of live (green) moss and increases with the amount of detritus in the sample — only 50 mL of raw material may be required to make a pellet if the moss is dense, clean, and green whereas as much as 250 mL may be needed for moss in the opposite state.

### Laboratory protocol

Moss samples were prepared for elemental analysis by cleaning, sorting, drying, grinding, and pelletizing each sample prior to XRF analysis. At every step, moss samples were manipulated with instruments and containers that were free of the metals investigated here: plastic petri dishes and forceps, scissors with ceramic blades, powder-free nitrile gloves, pure-aluminum oven dishes. All instruments were washed with soap and rinsed with 95% ethanol and distilled water between uses.

Cleaning of the moss samples involved removing dead tissue, bark, insects, and attached litter from the surface of the moss with sterilized plastic forceps. No washing was performed as it is ineffective at removing debris [2] and can remove deposited dust.

Differences in metal concentrations have been reported between basal and apical parts of moss shoots, for shoots of different lengths, and between older (usually darker green or green-brownish) and younger (green) moss pseudo-tissue, with additional variations among species [1,8]. Here all green parts were used rather than a subset of the shoot (e.g. top 2/3 or 3–4 cm of the shoot, [1,10]), as growth differences among sites could affect the relationship between tissue age and length.

After cleaning and sorting, moss shoots were dried in aluminum dishes at 40°C for 24 h. Samples were then manually ground until homogeneous using unglazed mortars and pestles with the help of liquid nitrogen. The resulting powdered samples were weighed, placed in glass vials, and subsequently either pelletized for laboratory XRF assays or acid-digested for quantitation by ICP-OES. Note that moss material from all mats sampled on a given tree were mixed to average out microvariations in moss exposure to pollution across the surface of tree trunks.

To make pellets, 0.5 g of powdered sample was placed in a 13 mm diameter evacuable pellet die (P/N GS03000, Specac, UK), which was pressed with 7 t for one minute using a manual 15 ton hydraulic press (Atlas™ model, P/N GS15011, Specac, UK). Binding powder was not added to the samples prior to this step as pellets made of pure moss did not crack (as long as pressure build-up and release was gradual over ~10 s).

Immediately after pressing them, the pellets were released from the die and placed on top of the pXRF instrument nozzle for measurement. The pXRF analyzer was placed in benchtop position with a protective lead covering, an Ultralene gridded window, and directly connected to a laptop. At every step, the pellets were handled with powder-free nitrile gloves by holding them on the sides of the disk. Note that both grinding and pellet making can be partly automatized to further shorten processing time.

An XRF assay was taken from each side of the pellets (to average out the potential impact of matrix configuration on a given side). Spectra were acquired for 120 s (~110 s live time) per assay using a voltage of 40 keV, an anode current of 25 µA and no vacuum. Pellets were subsequently kept in desiccators with gypsum (calcium sulfate) grains.

### Analytical chemistry

We measured the concentration of 23 elements by ICP-OES in each powdered moss sample: Al, As, B, Ba, Ca, Cd, Cr, Cu, Fe, K, Mg, Mn, Mo, Na, Ni, P, Pb, S, Se, Sr, Si, Ti, and Zn. Samples were pre-processed and analyzed by the University of Washington Soil Analytics Lab and Analytical Service Center (certified by the Washington State Department of Ecology) following standard protocols (Digestion: EPA 3050 & 3051; ICP-OES: EPA 200.7). For each assay, 1.5 g of powdered material was required — counting 0.5 g for each assay and allowing 1 g for replicates and potential errors. HNO_3_ + H_2_O_2_ digestion was used to prepare the samples; 4 mL of concentrated reagent-grade HNO_3_ was added to samples in 50 mL borosilicate tubes. The tubes were covered with plastic watch glasses and sat overnight at ambient temperature in a fume hood (approximately 18 °C) to allow some initial oxidation of the samples by the HNO_3._ Samples were digested at 95 °C for 90 min in borosilicate tubes heated evenly in a 36-tube graphite block digester. Samples were allowed to cool, after which 4 mL of reagent-grade 30% H_2_O_2_ were added to each tube followed by a 30-minute digestion at 95 °C and time to cool. Next, an additional 4 mL aliquot of H_2_O_2_ was added to each sample, which was heated again at 95 °C. After cooling, deionized water was added to each tube to the 20-mL mark. To remove any undigested particulates not dissolved in the HNO_3_ and H_2_O_2_, samples were filtered through 0.45-µm membrane syringe filters. Digests were then analyzed using ICP-OES (iCAP 6300, Thermo-Scientific, MA, USA).

### Data post-processing

**Fig. 2:**
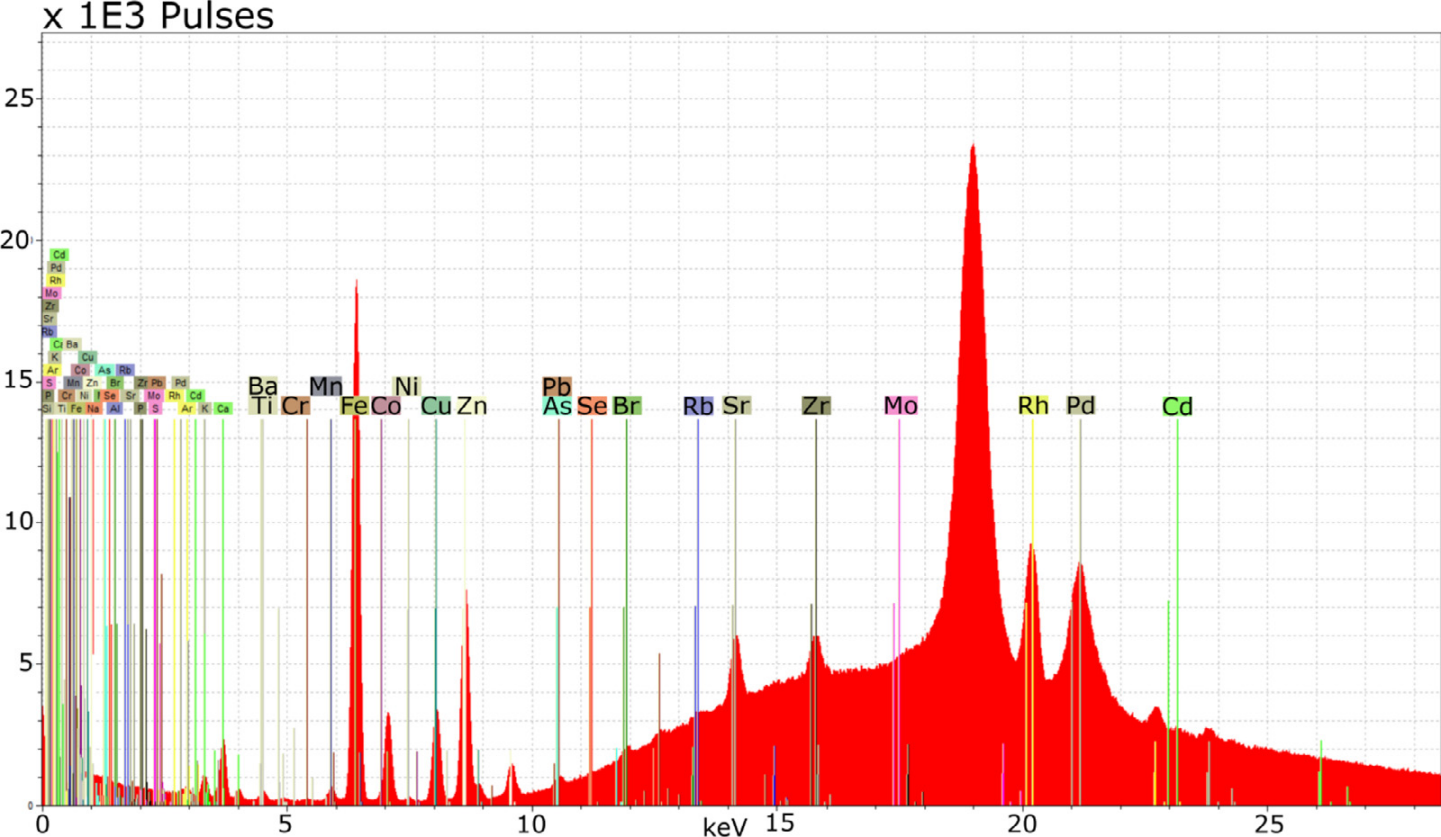
Screenshot of typical spectrum processing environment. The x-axis indicates kiloelectronvolts (keV) while the y-axis values are the total photon counts per channel (each channel capturing the number of pulses across 20.00–20.05 eV). Labels show the position of element-specific characteristic fluorescence peaks identified by the user. User-assisted Bayesian deconvolution extracts the magnitude of the fluorescence peaks above background counts, thus yielding an estimate of net photon count [20].

Spectral data from both *in-situ* and laboratory XRF assays were processed with standard tools in Bruker Spectra software 7.4.0.0 (Fig. 2; Bruker AXS Microanalysis GmbH, Germany) using Bayesian deconvolution, which corrects for background counts, escape peaks, sum peaks, Rayleigh scattering, and simple elemental overlaps. The output of this analysis is the computation of net photon count rates per second for each chemical element detected in the spectrum [20] — see https://www.xrf.guru/ for tutorials. To compare the spectral information among samples, the net photon count rate was summed across energy lines for each element; this sum was then normalized against the net count rate of the Rhodium-Compton peak (inelastic scatter from the instrument's tube), yielding what is hereafter referred to as ‘normalized count’ for the sake of simplicity.

### Data analysis

We assessed the applicability of the XRF-based methods for measuring metal concentrations in epiphytic moss based on the signal-to-noise ratio, the within-sample and within-site variability of measurements, and developed validation/calibration models using mass fractions determined by ICP-OES. Signal-to-noise ratio was assessed as the ratio between net and background photon counts obtained through the deconvolution procedure, averaged across all assays for each element.

The average within-sample and within-site coefficient of variations (*CV*) were measured for each element as:

(1) $$CV=100\frac{\sum_{i=1}^{N}\left(s/\bar{x}\right)}{N}$$

Where *s* is the sample standard deviation of metal concentrations and $\bar{x}$ is the sample mean. A coefficient of variation of 10 means that the standard deviation is equal to 10% of the mean, a CV > 100 means that the standard deviation exceeds the mean.

Within-sample *CV* quantifies method-specific sources of variance; it is not directly comparable among methods:

- For *in-situ* XRF (*N* = 72), *s* and $\bar{x}$ (sample statistics) were computed for the three different assays (on different moss mats) for a given tree and denoted as XRF_in-situ_ CV_tree_. XRF_in-situ_ CV_tree_ therefore describes the average variability in measurements across moss mats on a given tree.
- For laboratory XRF (*N* = 74), sample statistics were computed for the assays made on each side of the moss pellet (after grinding and homogenization of material from all mats on a given tree) and denoted as XRF_lab_ CV_pellet_.
- For ICP-OES (*N* = 2), sample statistics were computed for replicate measurements (using two separate aliquots of ground moss from mats of the same tree) and denoted as ICP-OES CV_replic._.

Note that XRF_in-situ_ CV_tree_ is necessarily higher than for the other two methods as within-sample *CV* for the latter is determined after sample homogenization.

Within-site *CV* quantifies, for each method, the variance across trees at a given site and thus the consistency (or replicability) of each method in estimating metal pollution at a given site; it is directly comparable among methods. For each element and method, within-site measurement variability was measured as the average CV of measurements among adjacent trees within a site (*N* = 19). Therefore, XRF*_in-situ_*, XRF_lab,_ and ICP-OES measurements were first averaged across moss mats, pellet sides, and replicates, respectively, for each tree and element. Sample mean and standard deviation were then computed using the results for the two adjacent trees for each site and element.

We demonstrated the utility of each XRF method for measuring metal concentrations in moss by fitting regression models with ICP-OES-measured concentrations as a reference. Models were developed for a subset of metals (Cu, Pb, Zn) but additional models could easily be developed in the future. General linear models (GLM) were developed for every XRF method and element. Trees (rather than sites) were used as our unit of analysis such that measurements were first averaged for each tree across moss mats (for XRF*_in-situ_*), pellet sides (for XRF_lab_), and replicate aliquots (for ICP-OES) prior to model development.

For each model, the error distribution of the response variable and the function linking expected values to the explanatory variable (link function) were determined by examining univariate distribution plots of the response and predictor variables as well as diagnostic plots of residuals. We chose the family of error distribution (gamma or gaussian) and link function (identity or log) which minimized the corrected Akaike Information Criterion for small samples AICc [13] and yielded normally distributed and homoscedastic residuals. Outliers were identified and removed based on deleted studentized residuals, but performance statistics are reported for models both with and without outliers. Outlier removal and data transformation are standard practices in developing such calibration models [21].

Contrary to ordinary least-square regression, a GLM does not produce a direct measure of goodness-of-fit such as the R^2^. Nevertheless, for the sake of comparability to similar validation studies, model performance is reported with the mean pseudo-R^2^ (the square of the correlation between the observed and predicted outcomes) and the symmetric Mean Absolute Percentage Error, or sMAPE [3], from a 10-fold cross-validation (50 repetitions).

(2) $$\text{sMAPE}\;\left(\%\right)=\;\frac{100}{N}\sum_{i=1}^{N}\frac{\left|{\hat{y}}_{i}-y_{i}\right|}{\left|{\hat{y}}_{i}\right|+\left|y_{i}\right|}$$

The matrix-specific lower limit of detection (LOD) with field XRF was not determined here as no blank samples were analyzed. The primary goal of our study (see companion paper [18]) was instead to identify metal pollution hotspots for Cu, Pb, and Zn. Nevertheless, minimum concentrations, LOD, and LOQ determined by ICP-OES are reported in Table 2.

### Method validation

**Fig. 3:**
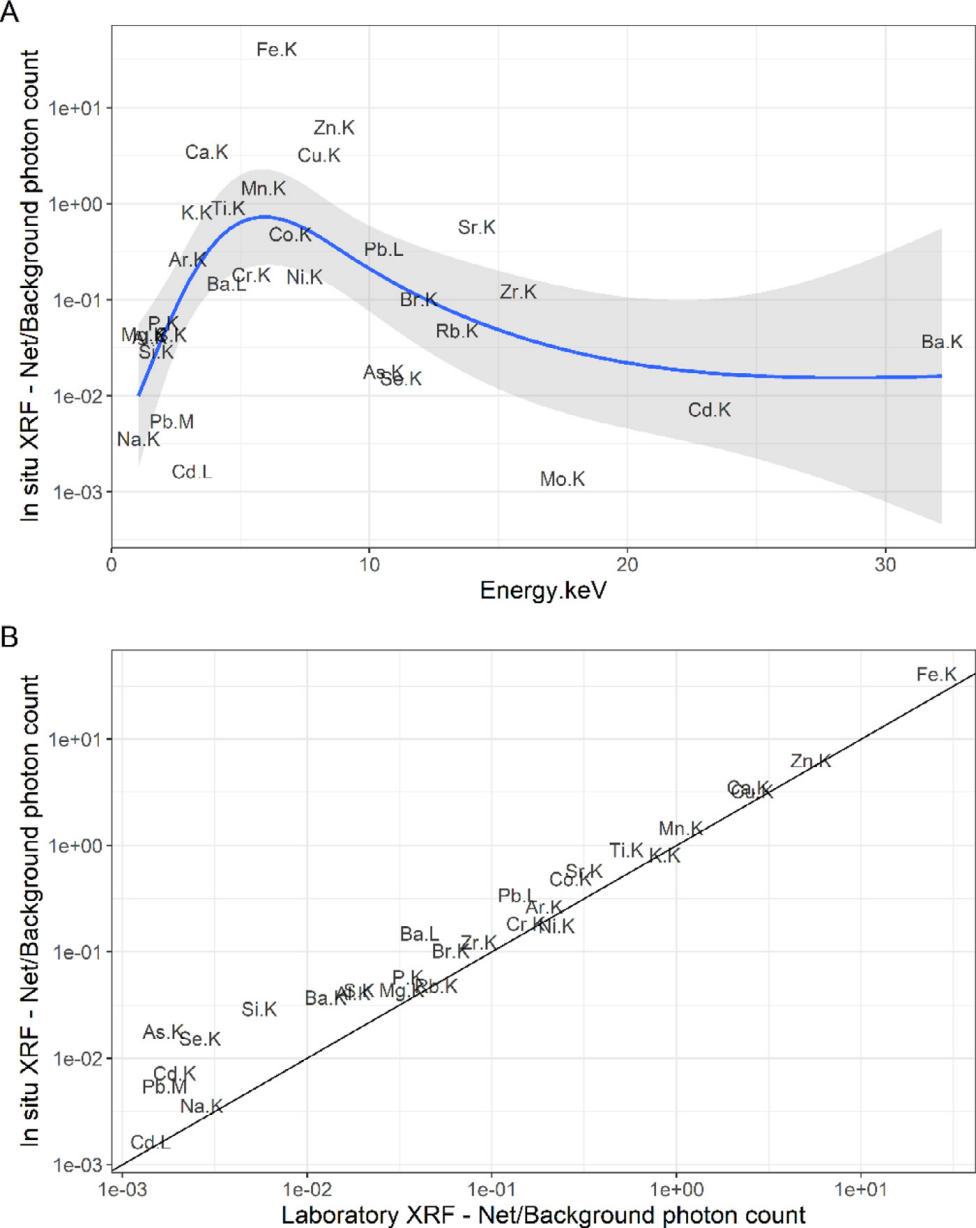
(A) Average ratio of net to background photon count per second (signal-to-noise ratio) for K- and L-shell emission peaks of detected elements for in-situ XRF and (B) signal-to-noise ratio comparison between in-situ and laboratory XRF measurements. The blue line (A) is the mean prediction of a LOESS fit (the gray ribbon is the 95% confidence interval) while the black line (B) is a 1:1 line.

For both XRF methods, the signal-to-noise ratio was highest for the elements whose absorption edge was within the energy range that was targeted by our measurement conditions (Fig. 3) — yellow excitation filter, 40 keV voltage, and an anode current of 25 µA. The signal-to-noise ratio was higher for laboratory XRF than in-situ XRF, which is due to the greater density and homogeneity of the matrix in the prepared moss pellets. Within-site variability of in-situ XRF was commensurate to that for ICP-OES measurements for e.g. Fe, Pb, Sr, Ti, and often even lower than that for laboratory XRF and ICP-OES reference concentrations, for e.g. Cr, Cu, Ni, Zn (Table 1), indicating that field-based XRF measurements exhibit the same level of replicability as more established methods. As expected by the patterns in signal-to-noise ratio, within-sample and within-site variability were low for elements whose absorption edge fell within our targeted window and those elements present at high concentrations in the samples — this pattern was evident across all methods. As, Cd, Mo, and Se were present at such low concentrations that even small changes in concentrations among measurements led to high CV_within-sample_ and CV_within-sample_. Lighter elements such as Al, Mg, Na, S, and Si, also exhibit high variability. The range of concentrations examined for Cu, Pb, Zn spanned 3 orders of magnitude (e.g., 1 – 300 mg kg^−1^) so a log-link was selected for all models.

**Table 1:** Summary table of within-sample variability, within-site variability, and mass fraction for all elements detected either with XRF methods or through elemental analysis by ICP-OES.

							Mass fraction (ICP-OES, mg kg^−1^)	
Element	XRF*_in-situ_* CV_tree_	XRF_lab_ CV_pellet_	ICP-OES CV_replic._ǂ	XRF*_in-situ_* CV_site_	XRF_lab_ CV_site_	ICP-OES CV_site_	Mean (min, max)	LOD | LOQ §
**Al**	63	62	4	27	40	18	1120 (327–2902)	0.011 | 0.037
**As**	130	131	–	77	124	141	< LOD (LOD-4)	0.004 | 0.013
**B**†	–	–	3	–	–	41	19 (0–59)	0.007 | 0.023
**Ba**	49	56	1	22	42	18	52 (17–129)	0.002 | 0.007
Br*	33	12	–	20	19	–	-	–
**Ca**	17	3	1	13	12	11	4312 (2114–6939)	0.014 | 0.047
**Cd**	82	81	–	37	56	141	< LOD (LOD-4)	0.001 | 0.003
Co*	61	39	–	25	44	–	-	–
**Cr**	35	18	5	16	35	37	6 (0–29)	0.003 | 0.010
**Cu**	14	2	2	10	12	22	27 (4–128)	0.005 | 0.017
**Fe**	27	3	1	20	18	20	1313 (310–5330)	0.005 | 0.017
**K**	24	5	1	26	25	16	4973 (2420–7453)	0.072 | 0.240
**Mg**	55	38	1	19	35	15	1645 (819–2851)	0.004 | 0.013
**Mn**	27	5	2	25	16	16	86 (32–254)	0.003 | 0.010
**Mo**	134	128	–	87	130	105	2 (LOD-27)	0.004 | 0.013
**Na**	71	62	10	69	75	68	708 (60–2152)	0.030 | 0.100
**Ni**	33	13	14	17	29	37	3 (LOD-8)	0.003 | 0.010
**P**	47	39	1	36	32	21	2055 (707–3607)	0.012 | 0.040
**Pb**	40	17	–	29	22	28	7 (LOD-61)	0.004 | 0.013
Rb*	30	8	–	19	15	–	-	–
**S**	80	68	2	35	47	17	1131 (546–2060)	0.004 | 0.013
**Se**	102	77	–	47	99	141	< LOD (LOD-4)	0.002 | 0.007
**Si**	78	82	2	44	61	24	176 (53–472)	0.003 | 0.010
**Sr**	15	3	3	12	11	10	35 (15–73)	0.003 | 0.007
**Ti**	25	8	2	18	11	20	49 (8–242)	0.005 | 0.017
**Zn**	15	2	2	11	14	19	92 (17–334)	0.029 | 0.097
Zr*	18	4	–	11	15	–	-	–

ǂ Elements marked with a - that were quantitated using ICP-OES were not detected in the replicate samples.

§ Limit Of Detection (LOD) and Limit of Quantification (LOQ).

⁎ The element was not quantitated using ICP-OES.

† The element was not detected using XRF analysis.

**Table 2:** Summary table of X-ray fluorescence calibration with General Linear Models (GLM) using mass fractions determined by ICP-OES as reference for Cu, Pb, and Zn.

	Response | Predictor	pseudo-R2 (w/ outliers)	sMAPE%	Equation*	GLM Family | link	N (outliers)
**Cu**	ICP-OES|XRF_in-situ_	0.75 (0.70)	36 (39)	5.28 + 3.42 log_10_ XRF_in-situ_	Gamma | log	70 (2)
**Pb**	ICP-OES|XRF_in-situ_	0.63 (0.61)	39 (39)	4.06 + 1.85 log_10_ XRF_in-situ_	Gamma | log	57 (1)
**Zn**	ICP-OES|XRF_in-situ_	0.81 (0.71)	23 (29)	5.04 + 2.50 log_10_ XRF_in-situ_	Gamma | log	69 (3)
**Cu**	ICP-OES|XRF_lab_	0.96	17	2.96 + 6.57 log_10_ XRF_lab_ - 1.15 (log_10_ XRF_lab_) ^2^	Gamma | log	74
**Pb**	ICP-OES|XRF_lab_	0.92	19	5.47 + 2.51 log_10_ XRF_lab_	Gaussian | log	59
**Zn**	ICP-OES|XRF_lab_	0.97	10	5.29 + 2.65 log_10_ XRF_lab_	Gamma | log	74

⁎ *p* < 0.001 for all coefficients.

The U.S. Environmental Protection Agency (USEPA) establishes three data quality levels for XRF measurements: (1) definitive, (2) quantitative screening, and (3) qualitative screening based on explained variance (R^2^) from the regression between XRF measurements and reference data — (1) R^2^ = 0.85 to 1.0, (2) R^2^ > 0.70, and (3) R^2^ ≤ 0.70 —, a paired *t*-test (on log_10_-transformed data), and the relative standard deviation [12]. Although we do not provide such a formal assessment, here we assess the quality of our model fits based on the same R^2^ criterion.

**Fig. 4:**
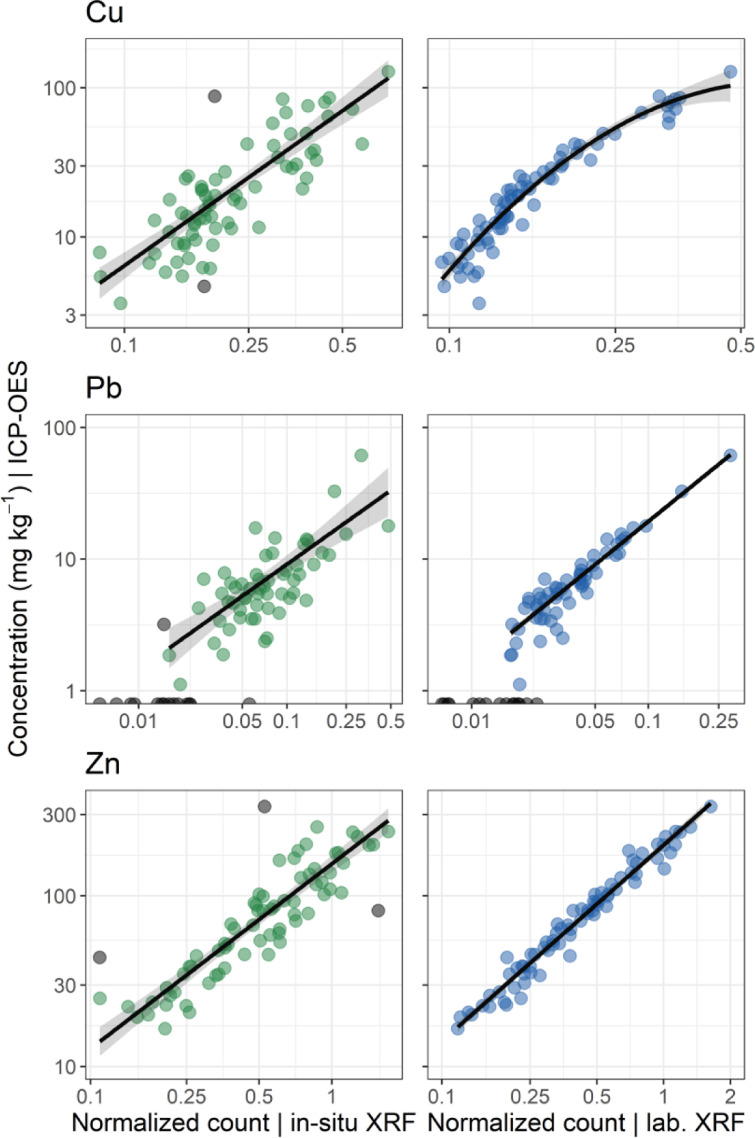
In-situ and laboratory (lab.) XRF calibration model fits using ICP-OES-measured concentrations as reference for Cu, Pb, and Zn. Each point's position represents the XRF and ICP-OES measurements for a given tree (averaged across moss mats and pellet sides for in-situ and laboratory XRF, respectively). gray points show outliers that were removed for calibration model development based on deleted studentized residuals. For Pb, gray points intersecting with the x-axis show samples for which concentrations measured by ICP-OES were reported as either Not Detected (ND, if < LOD) or TRace (TR, if <LOQ). See Table 2 for details on model fits. The black line and gray ribbon represent the mean and 95% confidence interval, respectively, of the predicted concentration for each XRF value. All axes are log-transformed.

In terms of explained variance, our assessments show that *in-situ* XRF enables qualitative screening of Pb (pseudo-R^2^ = 0.63) and quantitative screening of Cu (pseudo-R^2^ = 0.75) and Zn (pseudo-R^2^ = 0.81). Accordingly, it is recommended that at least 10 percent of the screening data be confirmed using analytical methods, QA/QC procedures and criteria associated with definitive data [12]. Laboratory XRF on the other hand, provides definitive data quality for all three elements (R^2^ > 0.90). Samples for which Pb concentrations measured by ICP-OES were below the limit of quantification (LOQ, *n* = 17) were excluded from the Pb calibration models (and shown as gray points intersecting with the x-axis on Fig. 4).

### Sources of uncertainty and improvement

Instrument precision is usually the least significant source of error in pXRF analysis; the main sources of variance in measurement include physical and chemical matrix effects, inconsistent positioning, depth, and moisture content of samples — for more details, see Kalnicky & Singhvi [14], Messager et al. [18], Towett et al. [20], and United States Environmental Protection Agency [21]. Since climatic factors can influence the temporal concentration of metals in epiphytic moss, it is usually recommended that either all sampling be conducted within a single season or that resampling be performed at the same sites across different periods of the year to capture seasonal variability [8]. We hence recommend that future studies assess the need for seasonal calibration models. In this study, short count times were used (60 s and 120 s for in-situ XRF and laboratory XRF, respectively) with the objective to demonstrate these techniques for a rapid sampling approach, but greater precision and sensitivity could be achieved by increasing count time. Further sensitivity analysis is warranted to fully characterize the uncertainty associated with these methods. One last limitation of XRF is that the prevalence of some commonly used tracers of non-exhaust traffic pollution (e.g. Sb, Ba; [6,^19^,23]) and other pollutants (e.g. Hg, V, Rh, Pd) cannot be accurately determined at the same time as those discussed in this study. These elements’ characteristic x-ray emission lines occur outside of the region that the instrument was set to measure (through the use of a yellow filter, see section *XRF Instrumentation*), resulting in insufficient signal-to-noise ratios for detection and/or quantification. See Bruker Elemental [4] for guidance on choosing adequate filter, current, and voltage settings based on target elements.

## Conclusion

Our validation shows that in-situ XRF enables qualitative screening of Pb and quantitative screening of Cu and Zn while laboratory XRF provides definitive quantitation for all three elements, which demonstrates that XRF analysis can be used for accurate and replicable moss biomonitoring of metal pollution. *In-situ* XRF is a non-intrusive and rapid (no sample processing and < 5 min measurement duration) approach which can be used as a first-level screening to assess the relative distribution of metal pollution across many sites, whereas laboratory XRF provides the highest level of data quality and can thus be used as a full replacement for more conventional elemental analysis techniques, provided that adequate calibration is performed beforehand. Implementation of these methods can thus drastically reduce the time and budget required for biomonitoring of metal pollution.

## Declaration of Competing Interest

The authors declare that they have no known competing financial interests or personal relationships that could have appeared to influence the work reported in this paper.
